# Intake of dehydrated nopal (*Opuntia ficus indica*) improves bone mineral density and calciuria in adult Mexican women

**DOI:** 10.3402/fnr.v57i0.19106

**Published:** 2013-05-21

**Authors:** María de los Angeles Aguilera-Barreiro, José Alberto Rivera-Márquez, Héctor Miguel Trujillo-Arriaga, Juan Alfredo Tamayo y Orozco, Eduardo Barreira-Mercado, Mario E Rodríguez-García

**Affiliations:** 1 Doctorado en Ciencias Biológicas y de la Salud, Universidad Autónoma Metropolitana, Delegación Coyoacán, D.F., México; 2 Facultad de Ciencias Naturales, Licenciatura en Nutrición, Universidad Autónoma de Querétaro, Juriquilla, Qro., México; 3 Departamento de Atención a la Salud, Universidad Autónoma Metropolitana Xochimilco, Delegación Coyoacán, D.F., México; 4 Departamento de Ingeniería Eléctrica, Área de Ingeniería Biomédica, Universidad Autónoma Metropolitana Ixtapalapa, Delegación Iztapalapa, D.F., México; 5 Comité Mexicano para la Osteoporosis, Delegación Álvaro Obregón, D.F., México; 6 Universidad del Valle de México, Campus Querétaro-Juriquilla, Boulevard, Juriquilla, QE, México; 7 Departamento de Nanotecnología, Centro de Física Aplicada y Tecnología Avanzada, Universidad Nacional Autónoma de México, Provincia Juriquilla, Qro., México

**Keywords:** calcium intake, bone mineral density, hypercalciuria, low bone mass, dehydrated nopal, osteoporosis

## Abstract

**Background:**

The intake of dehydrated nopal (DN) at a high stage of maturity along with high calcium content could improve bone mineral density (BMD) and calciuria and thus prevent osteoporosis.

**Objective:**

To evaluate the effect of calcium intake from a vegetable source (DN) on BMD and calciuria covering a 2-year period in menopausal and non-menopausal women with low bone mass (LBM).

**Methods:**

The study was quasi-experimental, blinded, and randomized, and included 131 Mexican women aged 35–55. Urinary calcium/creatinine index (CCI) was determined; BMD was analyzed on lumbar spine and total hip regions. Four groups were studied: Control group (CG), women with normocalciuria and a minimum dose of DN; experimental group 1 (EG1), women with hypercalciuria and a minimum dose of DN; experimental group 2 (EG2), women with hypercalciuria, and a maximum dose of DN; and normal group (NG) for reference in BMD.

**Results:**

After the first semester of treatment, calciuria levels in women from both experimental groups returned to normal, remaining constant for the rest of the treatment. The percentage difference in BMD increased in the total hip region in the CG (pre 4.5% and post 2.1%) and EG2 (pre 1.8% and post 2.5%) groups significantly in comparison to NG and EG1, which exhibited a significant decrease in their BMD. BMD increased only for the lumbar region in the EG2 group (premenopausal).

**Conclusion:**

The use of a vegetable calcium source such as nopal improves BMD in women with LBM in the total hip and lumbar spine regions principally in the premenopausal women, maintaining constant and normal calciuria levels.

Osteoporosis is a major public health problem among Mexican women aged 50 and above with a prevalence of 16% for osteoporosis and 57% for osteopenia reported in this population group (1). Fractures associated with low bone mass (LBM) represent a major economic problem for the family and the government. Unfortunately, the frequency of fractures among this population is expected to continue increasing due to longer life expectancy in adult women (2). Bone mineral density (BMD) can be measured by dual X-ray absorptiometry (DXA) at different anatomical regions such as total hip and both femoral neck and lumbar regions. On the other hand, a biochemical marker of bone resorption, which determines hypercalciuria, is the CCI in urine that complements the diagnostics. The measurement of calciuria provides essential information about the calcium balance (3).

Calcium intake is essential for the growth and maintenance of bone. In postmenopausal women, the requirement of calcium is 1300 mg/day (4). However, in developing countries, like Mexico, calcium intake from dairy products is limited, mainly due to their high costs. Instead, the consumption of beans, corn and vegetables like nopal, is most frequent and less expensive. The efficiency of calcium absorption from vegetable sources is similar to that from dairy products. It all depends on the concentration of calcium inhibitors such as oxalic acid, phytic acid or dietary fiber (5, 6). Park et al. (7) have found that the intake of vegetable sources of calcium was important to reduce osteoporosis levels and to increase the BMD in post-menopausal Korean women. Therefore, researchers are now increasingly interested in calcium bioavailability from vegetable sources, which may help to improve the calcium nutritional status and help prevent BMD loss.

Cramer (8) showed that a Ca/P ratio in the diet in the range 0.66–1 reduces BMD. On the other hand, the ideal Ca/P ratio for the development and maintenance of bones in adults is 1:1.5 (9). Although diet is supposed to be the best calcium source, consumption of calcium supplements is recommended under certain circumstances. Compared to dietary calcium, calcium citrate has a better response on biochemical markers of bone turnover (10). However, calcium citrate decreased bone resorption markers significantly more than calcium carbonate in post-menopausal women (11).

Nopal (*O. ficus indica*) has been widely used in the traditional Mexican diet. This vegetable is usually eaten at its first stages of maturity, called ‘nopalitos’ (40 days). According to Rodríguez-García et al. (12) and Hernández-Urbiola et al. (13), the physicochemical properties of nopal change as a function of maturity; they found that at high maturity stage when weight of the pads is 600 g corresponding to 135 days of harvest, dehydrated nopal (DN) obtained by vacuum technique contains 34.40 mg/g of calcium while the DN obtained from nopal pads of 40 days has only 17.95 mg/g of calcium.

As it is well known, nopal has antinutrimental compounds such as calcium oxalates, but Contreras-Padilla et al. (14) found that its concentration decreases with maturity, as more mature nopals contain less calcium oxalate (11.4 mg, 40 days, and 4.25 mg/g, 135 days) (12–15). When nopal is dehydrated, its nutrient contents per net weight increase, as does its shelf life. Dehydration also makes it easier to have daily nopal intake in high doses. Nopal is widely produced and consumed as an economical vegetable. Taking into account the aforementioned results, nopal can be an excellent dietary calcium supplement to include in the daily diet, in order to increase the calcium intake.

The increase of calcium content in the diet is an excellent alternative to reduce the risk of losses in BMD. There are other nutraceutical products (16–21); however, in all previous studies, there are no clinical or biological data related to the effect of nopal on BMD. This article evaluates the effect of calcium from a vegetable source such as DN at a high maturity stage (600 g pads), on BMD and the CCI, as a marker of bone resorption in both pre-menopausal and post menopausal women aged 35–55 years, during a 2-year follow-up, with the hypothesis that calcium from DN at high stage of maturity could improve or maintain BMD and reduce calciuria, taking as a reference a diet with dairy products.

## Materials and methods

### Subjects

A longitudinal, quasi-experimental, blinded, and randomized 2-year temporary study was carried out. The study included 190 women aged 35–55 years with LBM from a total population of 817 women from central Mexico. However, during the 2-year experiment, nine women (8) from EG1 and one from EG2 did not conclude the experiment due to the following causes: medical treatment for osteoporosis (2), some of them had no attachment to the treatment (3), incomplete studies like Ca/Cr or densitometry (4). Thus, 181 women finished the study. Informed consent was obtained from all patients. The inclusion criteria were: participants who were not pregnant or nursing during the time of study; none of the subjects were undergoing hormone replacement, using steroids, taking medication for bone metabolism, kidney or liver disease or taking vitamin supplements; participants were either premenopausal or menopausal for less than 10 years (either naturally or through surgical procedure); and finally women undergoing osteoporosis treatment or hormone therapy, taking supplements or no longer willing to participate in the study were eliminated. According to the aforementioned criteria, four groups were formed: normal group (NG) that included women with normal BMD as reference population by DXA (n=50), a control group (CG), including women with normocalciuria (n=66) and finally the two experimental groups with hypercalciuria (n=65), called experimental group 1 (EG1, n=29) and experimental group 2 (EG2, n=36) that were supplemented with DN. EG1 and EG2 were randomized using a C++ program.

### Physicochemical characterization of nopal and nopal doses

Nopal pads of *O. ficus indica var. redonda* were used in this study; the vacuum drying process has been reported elsewhere (12–14) and the physicochemical characterization of DN 600 g is reported in Table 1. It is clear that nopal used in this experiment contained an important amount of calcium without oxalates.

**Table 1:** Chemical composition of dehydrated nopal g/100 g (*O. Ficus indica*, Redonda variety)

Nutriment	Nopal 600 g
Protein (g/100 g)	7.07
Fat (g/100 g)	1.87
Soluble fiber (g/100 g)	9.8
Insoluble fiber (g/100 g)	56.8
Moisture (g/100 g)	4.18
Ash (g/100 g)	24.3
Calcium (mg/g)	35.3
Phosphorus (mg/g)	0.35
Zinc (mg/g)	0.08
Magnesium (mg/g)	9.55
Sodium (mg/g)	0.30
Calcium oxalate (mg/g)	3.4
Potassium (mg/g)	65
Calcium/phosphorus ratio	9.7

### Treatment

For this treatment, 181 participants were accepted in the trial. They all filled out a medical and nutrition questionnaire, and were provided with next-appointment reminder cards. Taking into account the 24-h recall, diet substitutes were given according to their energy requirements, including all the characteristics of a dietary regimen for osteoporosis.

The recommendations for the consumption of DN are added to the food or preferably mixed with water or natural juices. It is very important to recall that the mean daily value of calcium consumption (24-h recall) of the subject participants in this study was 648±124 mg/day, and that the calcium content of DN 600 g was 3.4 g/100 g. It was necessary to include the recommended 1300 mg/day (mainly from the dairy calcium) and a minimum dose of 2.5 g (67 mg Ca) of DN for CG and EG1, and 800 mg of calcium in the diet with 15 g to reach the recommended 500-mg dose as supplementation in EG2, in order to obtain 1300 mg/day (4). In the case of phosphorous, 1300 mg/day was recommended in order to obtain a Ca/P ratio equal to 1 (9). The complete diet formulation per group is shown in Table 2. For CG, EG1, and EG2, aerobic exercise was recommended such as walking for 30 min at least three times per week, and sun exposure at least 10–15 min/day. In order to determine the compliance of the treatment, there was a monthly follow-up, where women were questioned about the consumption of DN, diet, exercise, and sun exposure. Data were recorded in individual logbooks.

**Table 2:** Characteristics of the study groups

Group	Diet	Treatment
Reference population for DXA Normal Group (NG) n=55 Normal DXA	Delivers results and orientation. Without diet	Without treatment or controlled exercise and sun exposure
Control group (minimum dose) (CG) n=66 Low bone mass Without hypercalciuria	Ca dietary 1300 mg P dietary 1300 mg 400 IU vitamin D 75 mg vitamin C 300 mg magnesium 11 mg zinc 25 mg fiber 1 g protein/kg/day kilocalories to their requirement	Dehydrated Nopal (2.5 g) equivalent to 67 mg of calcium Aerobic exercise 30 min, 3 times/week Sun exposure from 10 to 15 min
Experimental group 1 (minimum dose) (EG1) n=29 Low bone mass Hypercalciuria ≥0.17 mg/mg	Same CG	Same CG
Experimental group 2 (maximum dose) (EG2) n=36 Low bone mass Hypercalciuria ≥0.17 mg/mg	Ca dietary 800 mg P dietary 1300 mg 400 IU vitamin D 75 mg vitamin C 300 mg magnesium 11 mg zinc 25 mg fiber 1 g protein/kg/day kilocalories to their requirement	Dehydrated Nopal (15 g) equivalent to 500 mg Aerobic exercise 30 min 3 times/week Sun exposure from 10 to 15 min

### BMD

BMD was evaluated at baseline between January and August 2008, and between January and August 2010 through DXA using a Hologic QDR Explorer series densitometer with a variation coefficient of 1.0% using the Hispanic data as reference (22). BMD was measured on the total hip and lumbar spine regions for the diagnosis of LBM; one or both areas were studied in accordance with the official position of the International Society for Clinical Densitometry (from −1.1 to −2.5 SD below the young reference mean) (23). Women with normal BMD meeting inclusion criteria were considered for the DXA reference population and labeled as NG. They were asked not to eat for 12 h prior to the study. Densitometry was performed at baseline and at an annual and 2-year follow-up.

### CCI studies

Urine samples were requested at a 2-h collection in the morning for the calcium and creatinine determination through the CCI (from the second morning urine collection and 2 h later, before 10:00 am). The determination of calcium and creatinine was performed using standard methods by photometry absorbance, using Roche Cobas Integra 400 plus software version 2.3 (Windows XP). Depending on the results, if the level of calciuria was ≥0.17 mg/mg, it was regarded as hypercalciuria (24, 25). The calcium/creatinine ratio was analyzed every 6 months during the 2-year study.

### Statistical test

Data were analyzed using SPSS Statistics 20.0. Continuous variables were expressed using mean values, whereas categorical variables were expressed using frequency values. Analyses of covariance were employed for testing differences between groups for calciuria and the percentage of difference between BMD_2010_−BMD _2008_ in the total hip, femoral neck, and lumbar spine regions included confounding factors covariates, such as age, high, lean mass, fat mass, and BMD basal. The analysis was done with pre-menopausal and post-menopausal women; a post hoc test was evaluated between group differences. P-values <0.05 were considered statistically significant.

## Ethics

All patients gave their informed consent before participation. This study was approved by the Ethics Commitment of the Medicine School of Universidad Autónoma de Querétaro (México).

## Results

### Subject characteristics

According to the screening process for the 817 women who were studied at the beginning of this experiment, 37.2% were diagnosed with LBM and 6.9% with osteoporosis. The prevalence of both LBM and osteoporosis in women aged 35–49 years was lower than in older women (30.7 and 2.3%, and 45.4 and 18.3%, respectively). Prevalence of both LBM and osteoporosis was higher in the lumbar spine than in the total hip (29 and 7% vs. 15 and 0%, respectively). Meanwhile, the prevalence in the femoral neck region was higher than in the hip (27 and 1%, respectively). The average age at diagnosis was 43 years, 46.6 years for LBM and 51.6 years in women with osteoporosis. The prevalence of hypercalciuria in women with LBM was 37%.

The general characteristics of the 181 women for study groups are given in Table 3.

**Table 3:** General characteristics of study groups (n=181)

Characteristics	NG (50)	CG (n=66)	EG1 (n=29)	EG2 (n=36)
Age basal, mean±SD, y	44.0±5.3^a^	46.2±5.8^ab^	46.6±5.7^b^	47.4±5.0^b^
Age basal 35–45, n(%), y	32 (64.0)	27 (40.9)	11 (37.9)	11 (30.6)
Age end 35–45, n(%), y	25 (50.0)	27 (40.9)	11 (37.9)	11 (30.6)
Age basal 46–55, n(%), y	18 (36.0)	39 (59.1)	18 (62.1)	25 (69.4)
Age end 46–57, n(%), y	25 (50.0)	47 (71.2)	19 (65.5)	29 (80.6)
Weight basal, mean±SD (kg)	66.0±5.3^a^	64.9±11.0^a^	64.6±9.8^a^	61.1±9.0^a^
Weight end, mean±SD (kg)	66.0±9.6^a^	64.2±11.9^a^	65.3±11.2^a^	60.9±9.2^a^
Height basal, mean±SD (cm)	155.0±5.0^a^	155.8±5.6^a^	155.0±5.9^a^	155.0±5.3^a^
BMI basal, mean±SD (kg/m^2^)	27.5±4.0^a^	26.6±4.7^a^	27.0±4.1^a^	25.3±3.2^a^
BMI end, mean±SD (kg/m^2^)	28.0±4.2^a^	26.4±4.8^a^	27.1±4.4^a^	25.0±3.5^a^
Lean mass basal, mean±SD (kg)	38.8±5.0^b^	37.7±4.9^ab^	37.2±4.9^ab^	36.0±4.2^a^
Lean mass end, mean±SD (kg)	38.7±5.0^a^	37.0±5.3^a^	37.6±6.4^a^	36.6±5.4^a^
Fat mass basal, mean±SD (kg)	25.4±5.5^a^	25.6±7.3^a^	25.5±5.8^a^	23.3±5.8^a^
Fat mass end, mean±SD (kg)	26.0±6.2 b*	25.3±7.8^ab^	25.9±6.0^b^	22.3±6.6^a^
Without menopause basal, n (%)	34 (68)	34 (51.5)	17 (58.6)	19 (51.4)
Without menopause end, n (%)	31 (62)	33 (50)	14 (48.2)	18 (48.6)
With menopause basal, n (%)	16 (32)	32 (48.5)	12 (41.4)	17 (48.6)
With menopause end, n (%)	19 (38)	33 (50)	15 (51.8)	18 (51.4)
Menopause age basal, mean±SD, y	45.3±5.4^a^	44.8±4.9^a^	47.2±3.2^a^	46.6±3.8^a^
Menopause age end, mean±SD, y	45.5±5.0^a^	45.1±5.2^a^	47.8±3.7^a^	46.7±3.8^a^
BMD lumbar spine basal, mean±SD (g/cm^2^)	1.046±0.09^a^	0.865±0.04^b^	0.857±0.05^b^	0.854±0.05^b^
BMD lumbar spine end, mean±SD (g/cm^2^)	1.041±0.10^a^*	0.851±0.06^b^*	0.835±0.07^b^*	0.843±0.06^b^
BMD total hip basal, mean±SD (g/cm^2^)	1.004±0.10^a^	0.866±0.09^b^	0.876±0.08^b^	0.840±0.08^b^
BMD total hip end, mean±SD (g/cm^2^)	1.003±0.10^a^	0.897±0.10^b^*	0.855±0.09^b^	0.844±0.10^b^*
BMD femoral neck basal, mean±SD (g/cm^2^)	0.900±0.09^a^	0.756±0.08^b^	0.748±0.09^b^	0.740±0.06^b^
BMD femoral neck end, mean±SD (g/cm^2^)	0.854±0.10^a^*	0.746±0.08^b^*	0.723±0.09^b^*	0.718±0.07^b^*

Data are mean±SD.

NG=normal group, CG=control group, EG1=experimental group 1, EG2=experimental group 2.

ab Values in the same row that do not share the same superscript letter are significantly different (analysis of variance, Tukey test, p<0.05).

* Values in the same column are significantly different (paired-samples T test, p<0.05).

These data correspond to basal and end characteristics of: age, weight, height, body mass index, lean mass, fat mass, as well as BMD for lumbar spine, total hip, and femoral neck. At the beginning of the study, the NG group shows significantly younger women than other groups. However, weight, height, and BMI did not differ significantly among the groups, and at the end of the trial, these variables follow the same behaviors. For lean mass, at the beginning of the study NG exhibits the highest value, but an important feature in the case of this parameter at the end of the study, this parameter remains constant for all groups during the 2-year experiment. It is well known that this parameter tends to decrease with age. Lean mass is considered as a protective factor for BMD. It means that diet as well as calcium contributed to the maintenance of lean mass in these women (26). Fat mass at the beginning of the experiments did not present significant differences among groups, but at the end of the study, the NG exhibits a significant increase in relation to EG2. This fact can be directly related to the following aspects: the follow-up of the diet, the increase in the fiber consumption (DN), exercise, and the treatment attachment. It is very important to recall that educational counseling about bone health was confirmed in the health of these women. In relation to the prevalence of menopause at the beginning of this study, NG showed 32% of prevalence, while this ranged from 41.4 to 48.6% for groups CG, EG1, and EG2. At the end of the study, the NG showed an increase of 6% (n=3), 2% (n=1) in CG in, 10% (n=3) in EG1, and 2% (n=1) in EG2. It means that only eight women presented menopause during the 2-year follow-up.

BMD shows the baseline BMD of the three studied regions, group by group, which demonstrated a correct diagnosis statistically and clinically. NG presents the BMD within normal ranges and BMD in the control groups and EG1 as well as EG2 show an LBM. It is interesting to observe, that for lumbar spine NG, CG, and EG1 the BMD decreased statistically, while EG2 did not show any statistical difference. In the case of total hip, NG and EG1 did not exhibit statistical differences, while CG and EG2 showed increases in this parameter. For the femoral neck region, all groups exhibited decreases in the BMD.

### Evaluation of calciuria in a five-semester follow-up

**Fig. 1:**
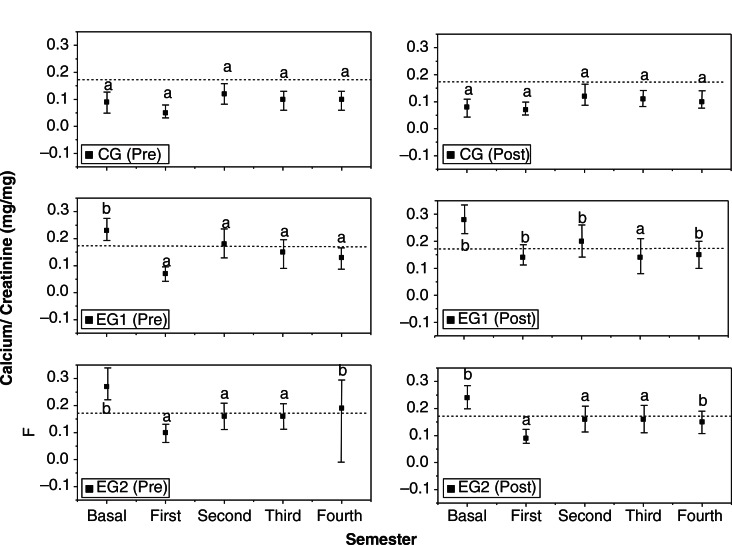
CCI (95% CI), per group and per semester, in pre- and post-menopausal women. CG (pre)=control group pre-menopausal women, CG (post)=control group post-menopausal women, EG1 (pre)=experimental group (minimum dose) pre-menopausal women, EG1 (post)=experimental group (minimum dose) post-menopausal women, EG2 (pre)=experimental group (maximum dose) pre-menopausal women, EG2 (post)=experimental group (maximum dose) post-menopausal women. ^ab^Data are mean 95% confidence interval (lower bound, upper bound), adjusted by age, BMD basal (lumbar spine, total hip and femoral neck), height, lean mass and fat mass. Values by groups that do not share the same superscript letter are significantly different (covariance, LSD test, p<0.05).

Figure 1 shows the Ca/Cr index of the different groups per semester. This analysis was done by taking into account menopause stage (pre- and postmenopause), by using analyses of covariance. The covariance was adjusted for the confounding factors: age, BMD basal (lumbar spine, total hip, and femoral neck), height, lean mass, and fat mass, with a post hoc test. Dashed lines in this figure indicate the hypercalciuria limit. It was possible to observe that at the first semester, that calciuria decreased in all study groups, but there were statistical differences in EG1 (post) without hypercalciuria; during the second semester, against EG1 (post) that exhibited hipercalciuria. From a clinic point of view, EG1 (pre) also exhibited hypercalciuria. At the third semester, it is important to observe that all groups remained normal (pre and post), with no statistical difference among them. In the fourth semester, EG1 (post), EG2 (pre and post) showed statistical differences in relation to CG (pre and post) and EG1 (pre). However, EG2 (pre) showed hypercalciuria.

### Evaluation of percentage difference in BMD at 2-year follow-up

**Fig. 2:**
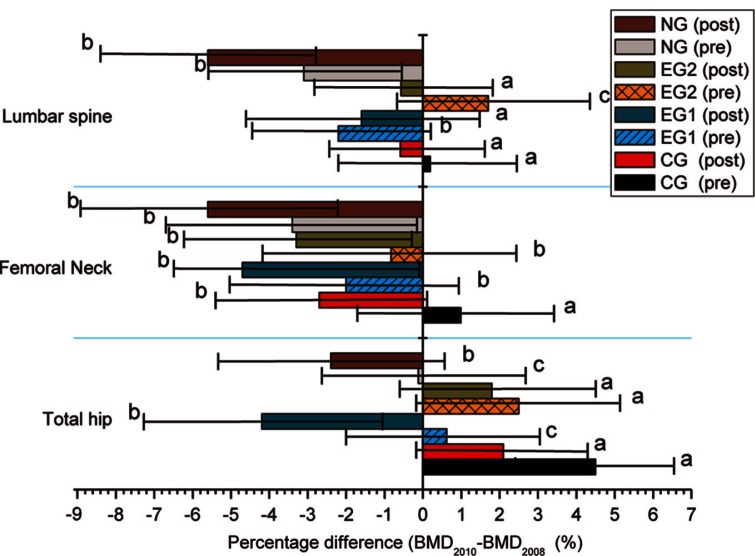
Percentage of the difference in BMD (BMD_2010_−BMD_2008_ (%)) for lumbar spine, femoral neck, and total hip; for pre- and post-menopausal women. CG (pre)=control group pre-menopausal women, CG (post)=control group post-menopausal women, EG1 (pre)=experimental group (minimum dose) pre-menopausal women, EG1 (post)=experimental group (minimum dose) post-menopausal women, EG2 (pre)=experimental group (maximum dose) pre-menopausal women, EG2 (post)=experimental group (maximum dose) post-menopausal women. ^abc^Data are mean 95% confidence interval (lower bound, upper bound), adjusted by age, BMD basal (lumbar spine, total hip and femoral neck), height, lean mass and fat mass. Values by groups that do not share the same superscript letter are significantly different (covariance, LSD test, p<0.05).

Figure 2 shows the behaviors of lumbar spine, femoral neck, and total hip as a function of the BMD_2010_−BMD_2008_ (%) taking into account the menopausal stage (pre and post) using covariance analysis adjusted by age, basal BMD (lumbar spine, total hip and femoral neck), height, lean mass, and fat mass. In the case of lumbar spine there was a decrease in the BMD for all groups and only EG2 (pre) increased statistically. For femoral neck, all groups exhibited a decrease and only CG (pre) increased statistically. In the case of total hip, EG1 and NG showed a decrease in this parameter, and CG and EG2 (post and pre) showed an increase in the BMD.

## Discussion

It is well known that dietary calcium from vegetables is an excellent option to improve or maintain bone health. The chemical proximate analysis of 600 g nopal used in this study showed that it contains a high amount of calcium and a low calcium oxalated content, which makes it an important source of dietary calcium. DN is an excellent calcium source.

The prevalence of LBM and osteoporosis found in this study was lower in relation to the prevalence of LBM in Mexico for women older than 50 years (1). In the case of the prevalence by regions, total hip and lumbar spine regions in our study showed lower prevalence than the studies carried out in Mexico City (27–29). This fact can be explained in terms of the age, because the women in this experiment were younger than the national prevalence in Mexico. According to the data obtained for calcium intake in this study, the low calcium consumption that was 648±124 mg/day could be one of the main factors for LBM. It is well known that calcium from the daily diet is the best source for bone maintenance. On the other hand, these women were aged from 35 to 55 years with 46% of them in the menopause stage. The hormonal state is also fundamental for bone formation and maintenance.

According to the covariance analysis, the results of CCI showed (Fig. 1) that in the case of EG2 (maximum DN doses), this parameter exhibited normocalciuria, with an improvement in the post-menopausal women, while the EG1 with minimum doses of DN exhibited variations in the calciuria levels along the experiment. It could be related to the calcium source. In the case of EG1, the calcium intake included dairy products in the diet, while the increase in the calcium intake of the EG2 was achieved mainly with a vegetable like DN. These findings agreed with Park et al. (7), which showed that vegetable sources of calcium are the best source of calcium to increase the BMD in post-menopausal Korean women. The fluctuation in the values of Ca/Cr index during the experiment could be related to other factors, such as changes in the adherence to treatment in diet, and decreased consumption of caffeine, protein, sodium, and phosphorus (3). It is crucial to mention that the women were periodically informed about the importance of diet during the study. This fact strongly influenced the consumption of different products that are beneficial for bone formation. Changes in habits, such as sun exposure, and the consumption of vitamin D in the diet could help in the control of hypercalciuria.

For all regions, as can be observed, BMD continued with the same diagnosis, and the women did not develop osteoporosis. In the analysis of the percentage differences (BMD_2010_−BMD_2008_ (%)) in total hip region, this parameter was found to increase (P<0.05) between CG (pre and post) and EG2 (pre and post) groups. It is interesting to note that clinically, the higher gain was found for premenopausal women. This is indicative that the hormonal stage is playing an important role in the bone formation, and although these groups behave equal statistically, the hip region had the highest rate of benefit. This response is quite important, as the maximum dose showed a response as good as the CG without hypercalciuria that consumed lower doses of DN. The covariance analysis showed that the response obtained from the lumbar spine and femoral neck regions BMD_2010_−BMD_2008_ (%) showed a decrease, but no osteoporosis was reached for any group. It is very important to recall that in the case of lumbar spine, EG2 (pre) showed a gain in BMD.

It is known that after 40 years of age there is a negative balance in the BMD which is responsible for the physiological loss of bone mass (0.5/year), while in the post-menopause, it is reduced from 3 to 5% per year, especially the trabecular bone (lumbar spine) during the first 7 years, then the loss is from 0.75% per year (30). When bone loss occurs quickly, it can cause stiffness and strength reduction, leading to bone fracture. When the loss occurs slowly, it only causes bone weight loss (31).

During the premenopausal stage, women absorb 25% of the calcium consumed in the diet (32); this is reversible with hormone replacement therapy by the direct effect of estrogen on calcium transport in the gastrointestinal tract (33). Hence, it is very important to prevent the rapid loss of BMD due to hypercalciuria before establishing a menopause nutraceutical treatment such as the DN 500-mg dose of calcium supplement to meet calcium requirements. This can be achieved by including a recommended diet for osteoporosis, at least 30 min of aerobic exercise three times per week, sun for 10 min a day, and avoiding the overuse of substances such as caffeine, cigarettes, and alcohol.

In this study, it was found that calcium intake, especially calcium from a vegetable source, such as nopal, is an excellent nutraceutical to improve BMD. In conclusion, the maximum DN dose used in this experiment improves BMD in women with LBM with hypercalciuria in the total hip and lumbar spine mainly in premenopausal women, maintaining the calciuria at the normal levels.
